# Investigating Behavioral and Neuronal Changes in Adolescent Mice Following Prenatal Exposure to Electronic Cigarette (E-Cigarette) Vapor Containing Nicotine

**DOI:** 10.3390/brainsci13101417

**Published:** 2023-10-06

**Authors:** Alaa AlHarthi, Fawaz Alasmari, Shakir D. AlSharari, Nouf M. Alrasheed, Musaad A. Alshammari, Tahani K. Alshammari

**Affiliations:** 1 Pharmacology & Toxicology Graduate Program, Pharmacy College, King Saud University, Riyadh 11211, Saudi Arabia; 438203019@student.ksu.edu.sa; 2 Department of Pharmacology and Toxicology, College of Pharmacy, King Saud University, Riyadh 11362, Saudi Arabia; ffalasmari@ksu.edu.sa (F.A.); sdalsharari@ksu.edu.sa (S.D.A.); nrasheed@ksu.edu.sa (N.M.A.); malshammari@ksu.edu.sa (M.A.A.)

**Keywords:** electronic cigarettes, nicotine addiction, adolescence

## Abstract

A substantial percentage of pregnant smokers stop using traditional cigarettes and switch to alternative nicotine-related products such as e-cigarettes. Prenatal exposure to tobacco increases the risk of psychiatric disorders in children. Adolescence is a complex phase in which higher cognitive and emotional processes undergo maturation and refinement. In this study, we examined the behavioral and molecular effects of first-trimester prenatal exposure to e-cigarettes. Adult female mice were divided into normal air, vehicle, and 2.5%-nicotine-exposed groups. Our analyses indicated that the adolescents in the 2.5%-nicotine-exposed group exhibited a significant lack of normal digging behavior, elevated initial sucrose intake, and reduced recognition memory. Importantly, we identified a substantial level of nicotine self-administration in the 2.5%-nicotine-exposed group. At a molecular level, the mRNAs of metabotropic glutamate receptors and transporters in the nucleus accumbens were not altered. This previously undescribed work indicates that prenatal exposure to e-cigarettes might increase the risk of nicotine addiction during adolescence, reduce cognitive capacity, and alter normal adolescent behavior. The outcome will aid in translating research and assist healthcare practitioners in tackling addiction and mental issues caused by toxicological exposure. Further, it will inform relevant policymaking, such as recommended taxation, labeling e-cigarette devices with more detailed neurotoxic effects, and preventing their sale to pregnant women and adolescents.

## 1. Introduction

The electronic cigarette (e-cigarette) device was originally developed by Chinese pharmacist Hon Lik, who created it as an alternative to conventional smoking. However, since their introduction to the market, an increase in e-cigarette use among both smokers and non-smokers has been reported, with 9 million people currently using these products [1]. E-cigarettes are battery-driven devices (typically called vapes) that deliver nicotine aerosols at diverse concentrations. These contain propylene glycol and glycerin, along with variable flavoring agents. In general, e-cigarettes are considered safer than conventional tobacco [2]. The perception that they are a safer alternative to traditional cigarettes because of reduced exposure to hazardous chemicals and tar may lead some cigarette smokers to switch to e-cigarettes during pregnancy [3].

Nicotine is the primary psychoactive substance in e-cigarettes; its effect is exerted by activating and desensitizing nicotinic acetylcholine receptors (AChRs) [4]. These modulate the release of norepinephrine (NE) and serotonin (5-HT) in the developing brain [5] and GABAergic signaling [6]. Nicotine addiction is a problematic disorder [7]. Long-lasting maladaptive mechanisms predominantly mediate addiction. These involve complex functions such as motivation, self-control, decision-making, and refined neuronal circuitry [8].

A wealth of evidence highlights the role of the dopaminergic pathway in mediating these mechanisms. However, a systematic review indicated that reports from randomized controlled trials do not advocate targeting the dopaminergic system in the treatment of addiction [9]. This implies a lack of successful treatment or preventive measures for these lifelong changes. A previous report indicated that nicotine abstinence modulates group II metabotropic glutamate receptors (mGlu2/3 receptors) [10]. Moreover, the dysregulation of astroglial glutamate transporter GLT-1 is implicated in the complex mechanisms of addiction relapse [11]. In support of this, studies have linked the glutamatergic pathway to nicotine exposure [12,13,14].

Smoking during pregnancy is associated with serious later complications; however, the impact of e-cigarette usage during the first trimester of pregnancy is far from understood. To the best of our knowledge, only two studies have examined the effects of e-cigarettes during pregnancy and lactation. The first study examined the impact of e-cigarette exposure starting from 15 gestational days and lasting through the lactation stage. At the same time, pups were directly exposed to e-cigarette vapor [15]. The second study revealed that prenatal and early postnatal exposure to e-cigarettes is associated with cognitive impairment in adulthood [16]. Although these studies examined the impact of e-cigarettes during adulthood, the findings of both indicate that prenatal and early postnatal exposure to e-cigarettes leads to the emergence of neurobehavioral changes in adulthood.

The current study was undertaken to analyze the consequences of prenatal exposure to e-cigarettes during pregnancy in adolescent mice. The behavioral analysis included the use of sucrose neophobia to assess anxiety [17]; this assessment has previously been utilized in studies assessing nicotine intake [18] and withdrawal [19]. A marble-burying test assessed the offspring’s well-being and normal digging behavior [20] and has previously been used to examine the impact of prenatal nicotine exposure on the behavior of offspring and their emotional regulation [21] and the impact of exposure to e-cigarettes [22]. Novel object recognition was assessed and has previously been used to evaluate recognition memory [23], cognitive deficit, and impaired recognition memory following exposure to nicotine [24]. Additionally, an oral nicotine preference test was performed to estimate the offspring’s preference toward nicotine consumption [25].

**Figure 1:**
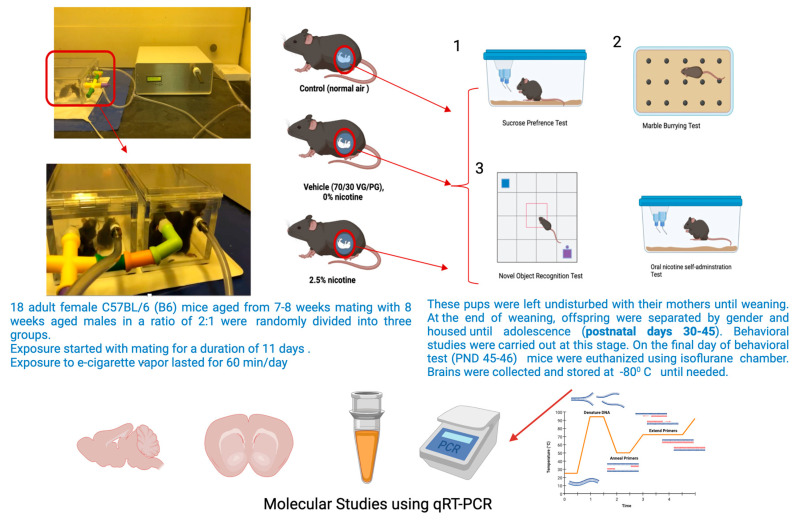
Schematic representation of the study design. Created using BioRender.

For these reasons, this project is significant and innovative and will aid in filling the gaps in knowledge regarding e-cigarettes and brain behavioral functions during that period. Our central hypothesis was that first-trimester prenatal exposure to e-cigarettes induces subsequent neurobehavioral changes that are a phenotypic signature of psychiatric disorders during adolescence. These might be mediated by glutamatergic connections. A schematic of the study design is presented in Figure 1.

## 2. Materials and Methods

### 2.1. Experimental Animals

A total of 18 adult (age 7–8 weeks) C57BL/6 (B6) female mice were obtained from the Animal Care Centre at the College of Pharmacy, King Saud University, Riyadh, Saudi Arabia. The mice were housed and maintained in environmentally controlled room temperature (22–24 °C) and humidity with a 12 h light/dark cycle and free access to water and food (regular chow). During pregnancy, nesting material was provided in home cages. All experimental procedures were performed in accordance with the guidelines of the King Saud University (Committee Reference #KSU-SE-20-67).

### 2.2. The Exposure Paradigm

An e-cigarette device was obtained from JUUL.com. A commercial e-cigarette vehicle solution composed of a 70/30 ratio of vegetable glycerin (VG)/propylene glycol (PG) which was berry-flavored was prepared in our laboratory with different concentrations of nicotine. A cigarette smoking machine (CSM-STEP) from CH Technologie (Westwood, NJ, USA), and (-) nicotine hydrogen tartrate salt powder from Sigma Aldrich (Burlington, MA, USA) (Cat#SLBD5910V) were also used. The exposure lasted 60 min/day for a period of 11 days. In terms of grouping, 3 dams were in the control and nicotine-exposed groups, and 2 dams were in the vehicle group.

### 2.3. Body Weight Tracking

The body weight of the pregnant female mice was measured twice weekly from the day of exposure until delivery and then two weeks after weaning. For the offspring, the body weight was measured after birth and at the beginning and end of adolescence, i.e., the timeframe in which the behavioral tests were carried out (PND30, PND45).

### 2.4. Sucrose Preference Test (SPT)

To examine anxiety-like behavior, we assessed neophobia to a novel taste (sucrose) upon first exposure. SPT was conducted as the first behavioral test for the offspring during adolescence. On postnatal days 32–35, the drinking water was removed for two hours from 8 to 10 a.m., and then two bottles were introduced to each mouse, one containing a 2% sucrose solution and the other containing drinking water. This test lasted for 30 min. Sucrose consumption was calculated as follows: sucrose consumption (mL)/(sucrose consumption (mL) + water consumption (mL)) × 100% [26].

### 2.5. Marble-Burying Test (MBT)

Some of the typical behaviors most rodent species exhibit are digging and burying. Therefore, we examined these features while performing marble-burying test tasks. We conducted this test two hours after the sucrose neophobia test, using 43 × 27 × 15 cm cages. Each mouse was placed separately in a cage with 20 marbles distributed equally across the mouse’s (sawdust) bedding at a depth of 5 cm. Thirty minutes later, we took images of the cages, and we calculated the number of buried marbles, considering whether more than 50% of the marble’s surface was covered by the bedding material [27].

### 2.6. Novel Object Recognition

This test was conducted to evaluate recognition memory and locomotor activity. The analysis was based on the ability of the mice to explore novel objects. The object recognition test was performed in a 30 × 20 × 13 cm box connected to ANY-MAZE software (Version 4.99 m). The test was divided into two sessions: a training session and a test session carried out one hour after the training session. During the training session, the animals were exposed to two identical objects (identical in size, form, and color) for 5 min; these objects were defined as familiar objects F1 and F2. One of the familiar objects was replaced by a new one (N) one hour after the training session, marking the start of the test session. The novel object was introduced so that the animals could explore the familiar object and the new one for a duration of 3 min. The animals were placed in front of the objects, facing the wall, at the beginning of each trial. The time taken to explore each object was recorded. A cross-over design was used in all test sessions whereby the new and familiar objects were alternately placed so as to exclude a potential preference for a particular spatial location of the objects. The term exploration is herein understood as smelling and touching the objects with the nose or with the forepaws and as the animal standing 2 cm or less away from the objects. The recognition index of each object was calculated. The time spent in the zone of each object was considered exploration time and was recorded by ANY-MAZE. The recognition index was computed as = (TN − TF)/(TF + TN) [TF = time used to explore the familiar object; TN = the time used to explore the new object]. The testing boxes used in the tests were cleaned with 70% alcohol between sessions [28]. The locomotor activity of the mice was recorded by tracking and measuring the total distance traveled during the testing phase and comparing this with the control group.

### 2.7. Oral Nicotine Preference Test

This test was performed to assess the offspring’s tendency toward nicotine preference. This test commenced during postnatal days 35–39 and lasted for the full six days of adolescence. The two-bottle method was used. Animals were singly housed in their home cage and presented with two fluid-filled bottles, one containing a 10% sucrose solution a (vehicle) and the other containing nicotine ± sucrose. The mice had access to food and drinking solutions for 24 h. The consumption data used were (1) the preference ratio (volume of drug-containing solution consumed ÷ total volume consumed) and (2) the drug dose consumed (volume ÷ concentration of drug solution [mg/mL] ÷ animal weight) [25]. The solutions were freshly prepared each day; to avoid side preferences, the bottles were switched on a daily basis, and food pellets were placed midway between the two bottles. In addition, two empty cages were filled with two bottles to calculate the loss of fluid due to leakage, handling, and evaporation. Consumed volumes in milliliters were measured daily using a measuring cylinder. When oral nicotine intake is measured, and because mice must be individually housed, single housing is a significant stress factor (social isolation stress). This can influence the results of oral self-administration studies. Therefore, in this experiment, the mice were already weaned, and an acclimation period of 1 day was allowed before the test was started the test. Studies using multiple nicotine concentrations (1–40 mg/L) have been particularly effective in detecting a genetic influence on oral nicotine intake. The nicotine concentration used in this experiment was 25 mg/L (25 mcg/mL) because 25 μL in saccharine can provide a nicotine consumption of up to 5 mL of nicotine on a daily basis [29]. Furthermore, the 25–30 μL concentration range induces more significant molecular effects [30]. Additionally, a different concentration consumption study reported that the amount consumed mostly came from a 25 μL concentration [31].

### 2.8. Molecular Studies: Quantitative Real-Time Polymerase Chain Reaction (qRT-PCR)

On day 7 after starting the nicotine preference test (at an age of 44–46 days), we sacrificed the animals. After anesthetizing the mice with isoflurane, a toe pinch was applied beforehand to ensure deep anesthesia. Next, the mice were decapitated, and their brains were isolated. The snap-frozen brains were then sectioned, and isolated sections of the nucleus accumbens were obtained via a cryostat. The total RNA was isolated from the nucleus accumbens from the right side of the brain. Next, qRT-PCR experiments were carried out by the Central Research Laboratory of the KSU female students’ campus. The protocol was as previously described [32]. RNA purification was conducted using the GeneJET RNA Purification Kit (Applied Biosystems; Thermo Fisher Scientific, Inc., catalog number K0731, Waltham, MA, USA). Following this, the samples were converted into cDNA using a High-Capacity cDNA Reverse Transcription kit (Applied Biosystems; Thermo Fisher Scientific, Inc., catalog number 4368814) in accordance with the manufacturer’s protocol. The RT-PCR was conducted using a SYBR Green detection system (SsoAdvanced Universal SYBR Green Supermix, BIO-RAD 172-5271) and performed using a ViiA 7 Real-Time PCR System. The thermal cycling was set up as follows: (1) a polymerase activation cycle at 95 °C for 30 s; (2) an amplification–denaturation cycle at 95 °C for 5–15 s; (3) 40 amplification–annealing/extension cycles at 95 °C for 15 s; and (4) a melt curve analysis at 60–95 °C for 2–5 s. The targeted genes were tested using the primers listed in Table 1. The relative level of expression of the target genes was analyzed using the quantitative comparative CT (∆∆CT) method, where the *GAPDH* CT value normalized the cycle threshold (CT) value of the targeted genes.

### 2.9. Statistical Analysis

The data presented in this work are expressed as the standard errors of means (SEMs). Statistical differences were computed using GraphPad Prism 9 software (GraphPad Software, San Diego, CA, USA). We performed a two-way ANOVA followed by Tukey’s multiple comparisons tests for the body-weight-tracking studies and daily nicotine consumption. We carried out a one-way ANOVA followed by Tukey’s multiple comparisons tests to analyze the sucrose neophobia, total fluid consumption, marble-burying test tasks, recognition memory, locomotor activity, and RT-PCR studies. Statistical differences were considered significant at * *p* < 0.05, ** *p* < 0.01, and *** *p* < 0.001.

## 3. Results

### 3.1. Body Weight Analyses of Adolescent Mice Following First-Trimester Prenatal Exposure to E-Cigarettes

**Figure 2:**
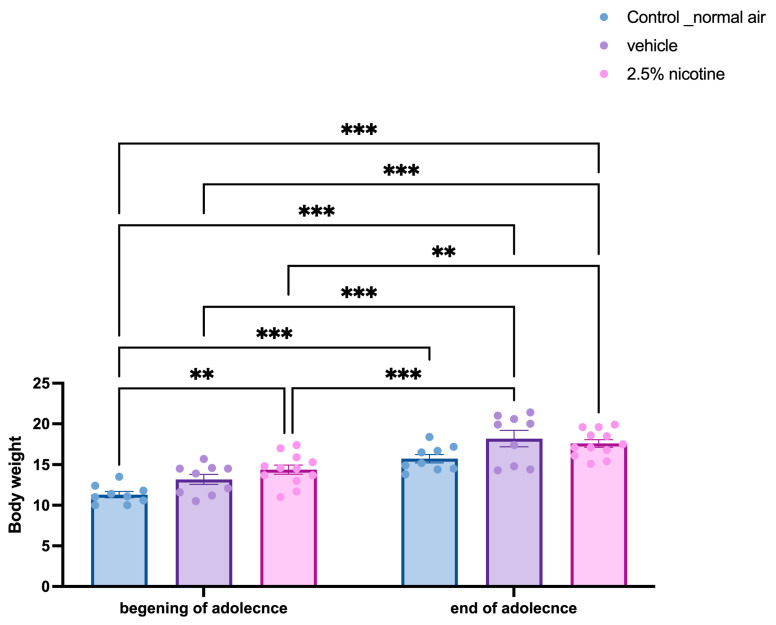
**Body weight analyses of adolescent mice following first-trimester prenatal exposure to e-cigarettes**. Bodyweight tracking in the adolescent mice at the beginning and end of adolescence in the tested groups. The body weight remained significantly increased throughout adolescence (*n* = 9 control, vehicle 9, and 12 mice in 2.5%-nicotine-exposed groups). Data were analyzed using a two-way ANOVA followed by Tukey’s multiple comparisons tests (data are presented as weight changes in grams (means + SEMs). Asterisk Significantly different when compared to controls. ** *p* < 0.01; *** *p* < 0.001.

Overall health was measured by tracking body weight. Figure 2 represents the effect of exposure to 2.5% nicotinic e-cigarettes compared to normal air and the vehicle (70/30 VG/PG) groups. Body weight was monitored throughout adolescence, starting at postnatal days 32–35 and ending at postnatal days 45–46. All groups exhibited a steady increase in body weight during the adolescent stage and at the beginning of adolescence versus the end of adolescence in the normal air (*p* < 0.01), vehicle (*p* < 0.01) and 2.5%-nicotine-exposed groups (*p* < 0.05). At the beginning of adolescence, the normal-air group displayed a significant difference in body weight readings compared to the end of the adolescence for both the vehicle and 2.5%-nicotine-exposed groups (*p* < 0.001). Similarly, at the beginning of adolescence, the vehicle group exhibited a significant difference in body weight readings compared to the end of adolescence for the 2.5% nicotinic group (*p* < 0.001). Notably, at the beginning of adolescence, the 2.5%-nicotinic-e-cigarettes group displayed a significant increase in body weight compared to the normal-air group (*p* < 0.001, Figure 2).

### 3.2. Sucrose Neophobia among Tested Groups

**Figure 3:**
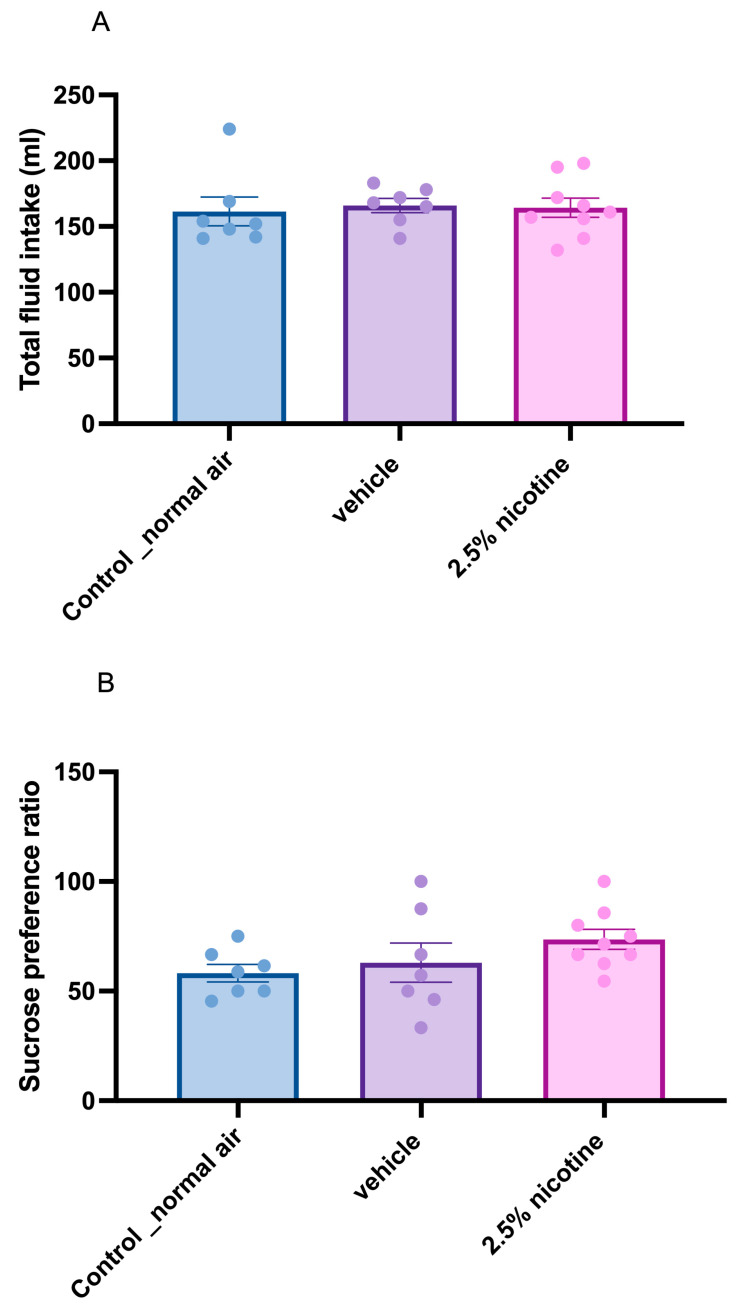
**Sucrose neophobia test among tested groups.** (**A**) Analysis of total fluid consumption among the tested groups in milliliters (*n* = 7 in the control and vehicle groups and 9 in the 2.5%-nicotine-exposed group). (**B**) Analysis of the amount of sucrose consumed—initial exposure—(*n* = 8 in control, 9 in vehicle, and 12 in 2.5%-nicotine-exposed groups). Data were analyzed using a one-way ANOVA followed by Tukey’s multiple comparisons tests (presented as means + SEMs).

Next, we assessed sucrose neophobia. First, we analyzed the quantity of total fluid consumed among the groups, using a one-way ANOVA followed by Tukey’s multiple comparisons test, which indicated no significant difference in total fluid consumption, as depicted in Figure 3A. Later, we utilized the same statistical tests to assess sucrose consumption upon first exposure (sucrose neophobia). Although our results were not significant, we identified a trend toward an elevated initial sucrose preference, as indicated in Figure 3B.

### 3.3. Marble-Burying Behavior

**Figure 4:**
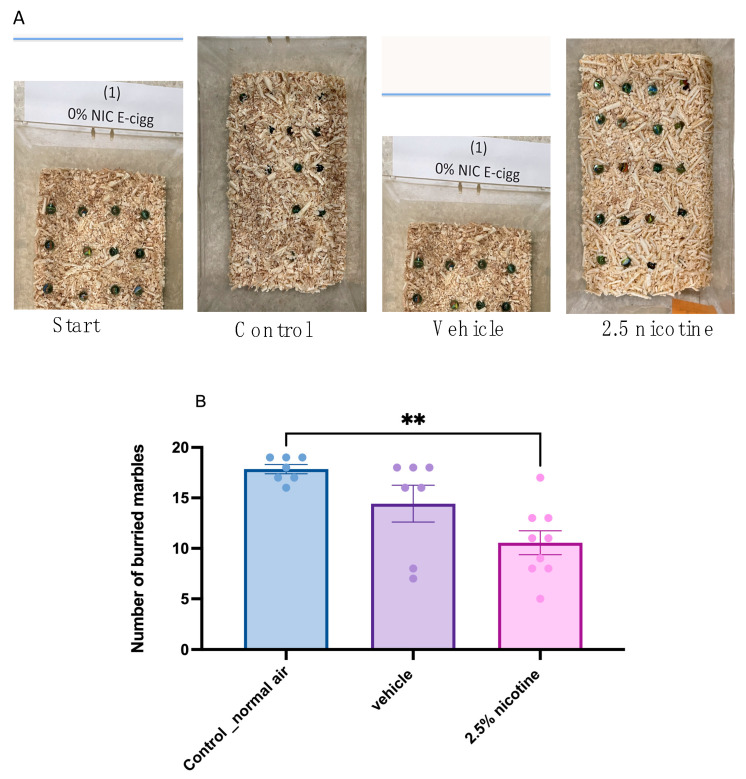
**Marble-burying behavior among tested groups.** (**A**) Representative images of the test at the beginning and after thirty min. (**B**) Quantification of the number of buried marbles in the tested groups (*n* = 7 in the control and vehicle groups and 9 in the 2.5%-nicotine-exposed group). Data were analyzed using a one-way ANOVA, followed by Tukey’s multiple comparisons tests (presented as means + SEMs). Asterisk Significantly different from the control, ** p < 0.01.

Some of the typical behaviors most rodent species exhibit are digging and burying. Therefore, we examined these features while performing marble-burying test tasks. Our results indicated that both the control and vehicle groups buried comparable numbers of marbles. However, the 2.5%-nicotinic-e-cigarette group exhibited a significant reduction in the number of buried marbles compared to the normal-air group (*p* < 0.01), as shown in Figure 4B.

### 3.4. Examining Recognition Memory Using a Novel Object Task

**Figure 5:**
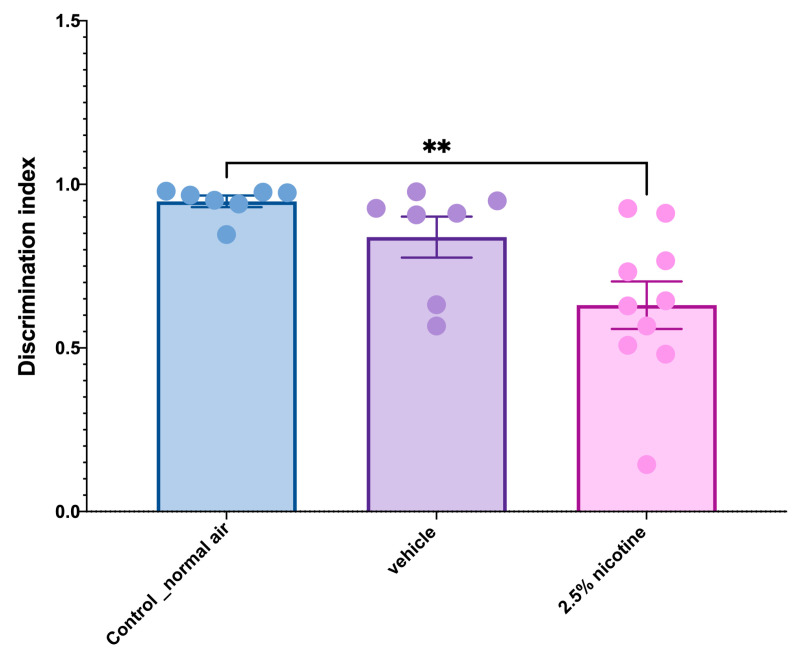
**Examining recognition memory while performing novel object tasks**. A discrimination index quantification using a one-way ANOVA followed by Tukey’s multiple comparisons tests (*n* = 7 in control and vehicle groups and 10 in the 2.5%-nicotine-exposed group) (data presented as means + SEMs). Asterisk Significantly different from controls, ** *p* < 0.01.

The analysis of recognition memory using a discrimination index as an end measurement revealed that the adolescent mice exposed to the 2.5% nicotinic e-cigarettes exhibited a significant reduction in recognition memory compared to the normal-air group (*p* < 0.01), as indicated in Figure 5.

### 3.5. Investigating Locomotor Activity while Performing Novel Object Tasks

**Figure 6:**
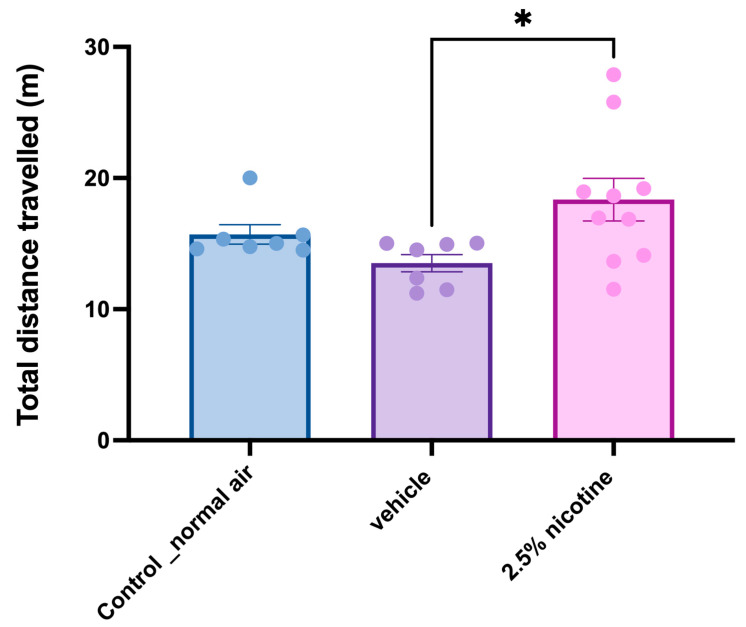
**Investigating locomotor activity while performing novel object tasks.** Locomotor activity quantified using a one-way ANOVA followed by Tukey’s multiple comparisons tests (*n* = 7 in the control and vehicle groups and 10 in the 2.5%-nicotine-exposed group) (data are presented as means + SEMs). Significantly different from controls, * *p* < 0.05.

The analysis of locomotor activity measured the total distance travelled. Our one-way analyses indicated that adolescent mice exposed to the 2.5% nicotinic e-cigarettes exhibited a significant elevation in distance travelled compared to the vehicle group (*p* < 0.05), as displayed in Figure 6.

### 3.6. Tracking Daily Nicotine Consumption Ratio among Tested Groups

**Figure 7:**
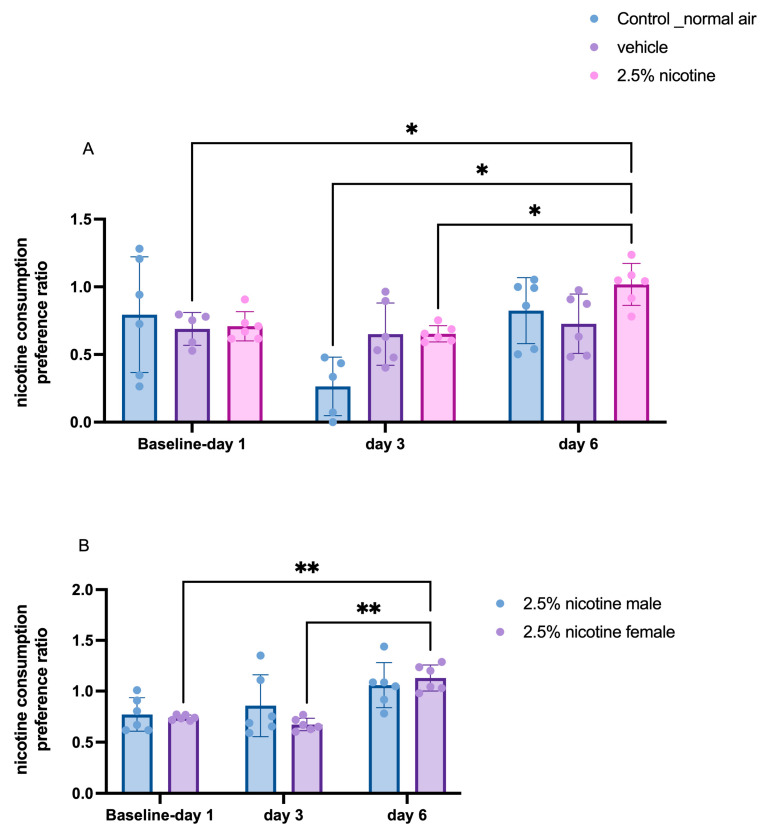
**Investigating nicotine consumption ratio.** (**A**) nicotine consumption ratio at three time-points, day 1, day 3, and day 6, using a repeated measures two-way ANOVA followed by Tukey’s multiple comparisons tests. (**B**) Quantification of nicotine consumption ratio in both genders in 2.5%-nicotine-exposed group using a two-way ANOVA followed by Tukey’s multiple comparisons tests (*n* = 6 mice per group). Data are presented as means + SEMs. Significantly different from the controls, * *p* < 0.05; ** *p* < 0.01.

For a period of six days, we tracked nicotine consumption. A repeated-measures two-way ANOVA followed by Tukey’s multiple comparisons tests indicated a significant difference in nicotine consumption at three time-points: day 1, day 3, and day 6. On the sixth day, the adolescent mice exposed to 2.5% nicotinic e-cigarettes exhibited a significant increase compared to the vehicle exposed group at day one, the normal-air group on day three, and the 2.5%-nicotine-exposed group at day three (*p* < 0.05). Next, we analyzed oral nicotine preference in the 2.5%-nicotine-exposed group using a gender-based two-way ANOVA. Our analysis indicated that on the sixth day, the adolescent female mice exposed to 2.5% nicotinic e-cigarettes exhibited a significant increase in oral nicotine preference compared to day one (*p* < 0.05) and day three (*p* < 0.01), as depicted in Figure 7.

### 3.7. Molecular Assessment of Metabotropic Glutamate Receptors and Trannsporter mRNA Expression in the Nucleus Accumbens

**Figure 8:**
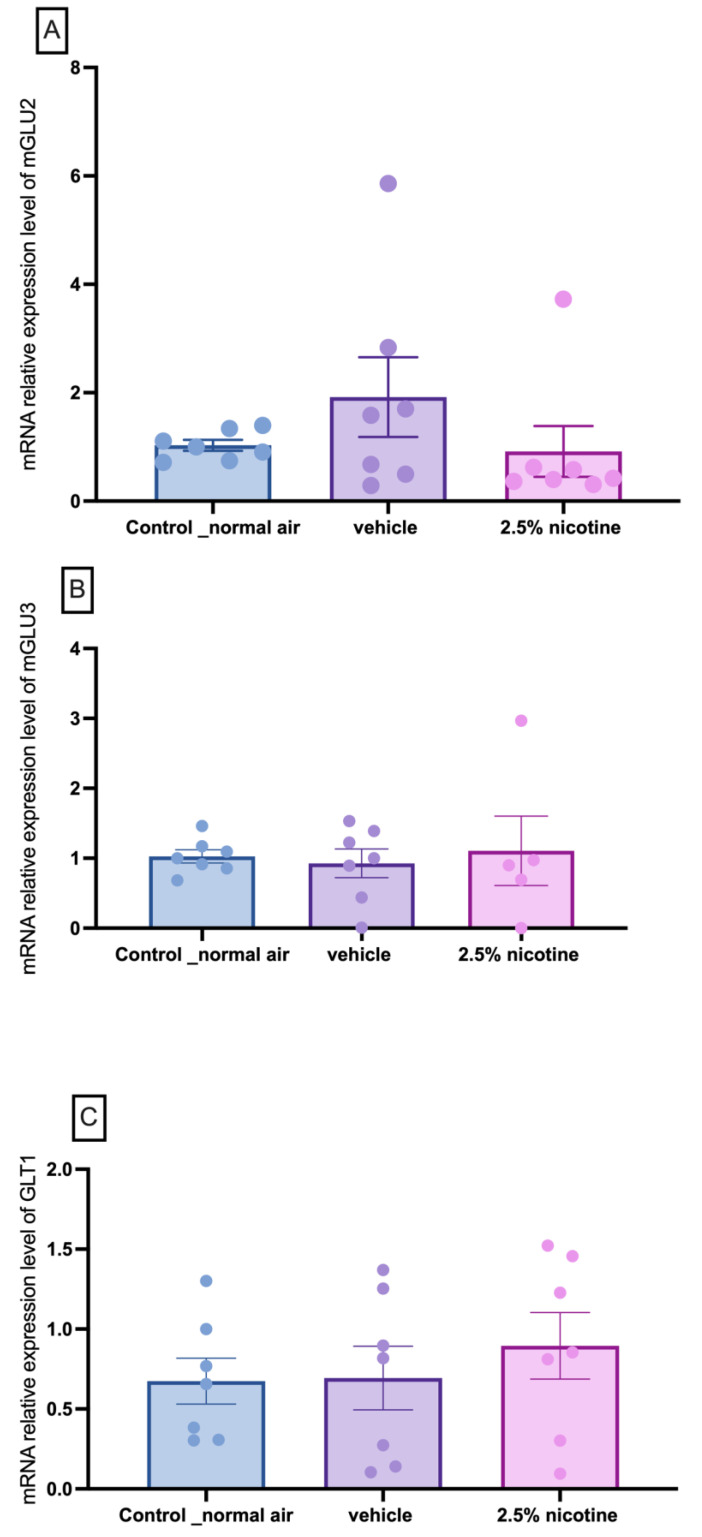
Molecular assessment of metabotropic glutamate receptors and transporter mRNA expression in the nucleus accumbens. (**A**) The mGlu2 mRNA expression level in the nucleus accumbens brain region was detected via an RT-PCR analysis. (**B**) The mGlu3 mRNA expression level in the nucleus accumbens brain region was detected via an RT-PCR analysis. (**C**) The GLT-1 mRNA expression level in the nucleus accumbens. The internal control was GAPDH. Data are expressed as a fold change and normalized to the control air-exposed group. The results are presented as means ± SEMs. The total number (N) comprises three to four biological replicants and two technical replicants. Data were analyzed using a one-way ANOVA followed by Tukey’s tests.

Following our behavioral assessments, we conducted molecular studies by postulating that these phenotypic alterations are linked to the Group II metabotropic glutamate receptors. To confirm this, we studied the mRNA expression levels of mGlu2, mGlu3, and GLT-1. A repeated measures two-way ANOVA followed by Tukey’s multiple comparisons tests indicated that the mRNA expression levels of mGlu2 mGlu3, and GLT-1 generated no detectable changes among the tested groups, as shown in Figure 8.

## 4. Discussion

In this work, we found that in comparison to the other groups, prenatal exposure to 2.5% nicotine elevated body weight, reduced the number of buried marbles, lowered the discrimination index, increased locomotor activity, enhanced nicotine preference, and revealed a trend of elevation in the mRNA expression levels of mGLU3 and GLT1 in the nucleus accumbens of the 2.5%-nicotine-exposed male group. Additionally, in the 2.5%-nicotine-exposed group, we observed a trend of elevated sucrose consumption while assessing sucrose neophobia.

The recorded weight gain indicated that the 2.5%-nicotine-exposed group exhibited the most significant gain in body weight from the beginning to the end of adolescence. Accumulating evidence has linked prenatal nicotine exposure and obesity later in life [33,34,35]. Clinical settings have revealed that prenatal exposure to traditional nicotine is associated with increased weight gain during adolescence, including subcutaneous and intra-abdominal fat [36]. In line with this, a review reported that prenatal exposure to nicotine is linked to elevated risks of obesity and metabolic dysfunction [37].

Our results revealed that the number of buried marbles was reduced in the 2.5 nicotine-exposed group, indicating that normal phenotypic digging behavior was altered. In a standard preclinical setting, marble-burying behavior was initially introduced to measure anxiety behavior, and then recommended as an indicator of repetitive obsessive-compulsive disorder behavior. It was later suggested as a way to model typical digging and burying behaviors [38]. Digging and burying are fundamental components of typical behavior in mice and rats and are considered a measure of rodents’ well-being [20]. This typical behavior is linked to the integrity of the hippocampal structure and function [39,40]. In a previous report on a transgenicmodel, the conditional deletion of transcription factor deformed epidermal autoregulatory factor 1 (Deaf1), and the reduced number of buried marbles was associated with a reduction in water maze contextual fear conditioning. Both tests are an index of hippocampal-dependent memory [41]. Additionally, in an animal model of Alzheimer’s disease (3xTg-AD mice) marble-burying was sensitive to aging and gender [42]. In another study of transgenic mice harboring alterations in the cyclin D2 gene, the hippocampal adult neurogenesis was disrupted, and the number of marbles buried was significantly reduced compared to wild type control mice [43]. In line with this, marble-burying and digging is interpreted as normal rodent behavior altered by targeting the hippocampal structure or function [44,45].

Another hippocampal-dependent function is recognition memory. Our data indicated a significant reduction in recognition memory. Nicotinic exposure during the early stages of adolescence is linked to decreased hippocampal-dependent memory, including working memory [46]. In another report, prenatal exposure to standard nicotine was linked to reduced cognitive capacity. One-month-old offspring mice exhibited reduced spatial working and object location memories while performing NOR tasks [47].

The observed reduction in recognition memory was not a function of reduced locomotor activity. In fact, our results revealed an elevation in locomotor activity in the 2.5 nicotine-exposed group. Previous reports indicate that prenatal nicotine administration to females in drinking water is linked to elevated locomotor activity in their offspring [48]. A previous study demonstrated that prenatal exposure to nicotine in drinking solutions increases locomotor activity in adult male offspring compared to females [49].

Our results indicated that the 2.5 nicotine-exposed group exhibited a pronounced tendency to consume nicotine on the sixth day of the nicotine preference test. Clinical and preclinical settings have linked prenatal exposure to nicotine and the tendency to consume nicotine later in life [50,51,52]. Furthermore, a previous study has shown that adolescents and females exhibit a more marked preference toward nicotine than males and adults [53]. Our results also indicated an altered pattern of nicotine intake between the third day and the sixth day. Notably, we observed a marked reduction in nicotine consumption in the control air-exposed group on the third day, but no reduction on the sixth day. We postulated that this could be attributed to post-ingestive factors. A previous study utilized DBA/2J (D2), C57BL/6J (B6), and A/J (A) mice strains, demonstrating that nicotine aversion was observable in most of these strains. Our results indicated that the 2.5%-nicotine-exposed group exhibited a pronounced tendency among the adolescent offspring to consume nicotine on the sixth day of the nicotine preference test at the age of 45–46 days. Clinical and preclinical settings have linked prenatal exposure to nicotine and the tendency to consume nicotine later in life [50,52,54]. Our data suggested that adolescent females exhibited a stronger tendency toward consuming nicotine than male mice. In support of our results, a previous study showed that adolescents and females display a more marked preference toward nicotine than males and adults [53].

Our molecular studies indicate that the mRNA levels of mGLU2, mGLU3, and GLT1 are not altered following prenatal exposure to e-cigarettes. However, we observed a trend in mGLU3 in the 2.5%-nicotine-exposed group. Our data indicate a trend of elevation in the mRNA levels of mGLU3 and GLT1 in the nucleus accumbens of the male group prenatally exposed to 2.5 percent nicotine. The opposite was observed in previous reports. Studies have revealed that chronic exposure to nicotine is linked to the downregulation of GLT-1 [12,14,55]. It is also of note that acute nicotine exposure is linked to the elevation of glutamate signaling in a GLT-1-mediated mechanism independent of dopamine and calcium signaling [56].

In another experimental setting composed of alcohol withdrawal and acute in vivo nicotine administration, the level of glutamatergic signaling was elevated in the core of the nucleus accumbens. The study comprised a complex design that included alcohol abstinence and acute nicotine exposure [57]. These studies indicated that the administration phase, whether acute or chronic, altered glutamate signaling, suggesting that coping and/or compensation mechanisms might be triggered.

The discrepancies in the expression of glutamate receptors between our results and a previous report could be attributed to multiple factors, such as the route of administration and animal species. It has also been reported that the stereotaxic implantation of nicotine into the nucleus accumbens of Sprague Dawley rats results in elevated glutamate levels.

Our study design was based on prenatal exposure to e-cigarettes. We postulated that prenatal neuronal programming might interfere with GLT1 expression. Pregnant females were exposed to 2.5% nicotine for the first ten gestational days. According to the Allen Brain Atlas, the expression levels of astroglia markers, including glial fibrillary acidic protein and S100, the neurotrophic calcium-binding protein, are at higher levels in the first ten days of pregnancy in the embryonic mouse brain than in other embryonic developmental stages. Later, in early postnatal stages, their levels of expression increase. This indicates that astroglia cells are moderately raised during the first trimester and the postnatal period [58], which may justify the trend toward elevated GLT-1 levels in our study as compared to other cells, astroglia cells predominately express GLT-1.

The impact of e-cigarette exposure has been extensively investigated at a molecular level in the nucleus accumbens. For example, in the context of nicotinic receptors, a previous report indicated that three weeks of e-cigarette exposure altered the mRNA expression of different nicotinic acetylcholine receptor subunits [59]. Similar findings were reported in a study in which nicotinic acetylcholine receptor expression was changed following one week of exposure to e-cigarettes [27]. In the context of the glutamatergic system, the functional link between the nucleus accumbens and the mechanism of addiction through glutametergic transmission was reviewed by Scofield and his colleagues [60]. They highlighted how glutamatergic synapses are implicated in mechanisms of addiction through the core and the shell. The glutamatergic afferents within the core are associated with reinstatement of drug seeking, while those within the shell are implicated in context-induced heroin seeking and relapse to cocaine seeking. Another study reported that exposure to e-cigarettes induces the downregulation of mGluR5 and GLT-1 in the nucleus accumbens shell but not the core [61]. In the current work, the technical incorporation of the nucleus accumbens included both the shell and the core (whole). These findings indicate that the glutamatergic system might be differentially involved within the core and shell of the nucleus accumbens. Future studies should therefore examine the impact of pre-natal exposure to e-cigarettes in the core of the nucleus accumbens versus its shell.

In terms of future work, we aim to study the effect of first-trimester conventional tobacco exposure on the tendency toward nicotine addiction in adolescent offspring, as previous preclinical studies were conducted either during the entre gestational period or in the third trimester only. Further, we aim to study the molecular effects of e-cigarette prenatal exposure on different brain regions, especially the hippocampus, as our data identified significant effects on hippocampal-dependent functions. We might further examine the cytoarchitecture of astroglial-related markers and examine their shape and arborizations. It is essential to track these differences in lifelong consequences to examine the effects of prenatal exposure on memory during the aging process.

Our findings suggest that the vehicle group behaves like the nicotine group. Previous studies reported that the e-cigarette’s base ingredients (propylene glycol and glycerin) were associated with multiple adverse effects ranging from eye and respiratory irritation to profound CNS effects, and it caused neurobehavioral manifestations [62,63].

This study reported for the first time the impact of first-trimester prenatal exposure to e-cigarettes, addressing an unmet need in this area of research. However, some limitations remain. For example, the sample size ranged from seven to nine, and even though previous reports utilized a similar sample size range sample size [64,65,66,67], it is considered relatively low. Another limitation is that prenatal nicotine exposure throughout pregnancy in mice indicates that prenatal exposure exhibits alterations in a sexually dimorphic pattern [68]. Future studies should therefore analyze gender differences in the impact of prenatal exposure to e-cigarettes.

## 5. Conclusions

This previously undescribed work indicates that prenatal exposure to e-cigarettes might increase the risk of nicotine addiction in offspring during adolescent stages, reduce cognitive capacity, alter normal adolescent behavior, and increase the risk of mood disorders in mothers. The study outcome may be aid in translating the research and assist healthcare practitioners in tackling issues associated with addiction and mental issues due to toxicological exposure. Further, it will inform relevant policymaking, such as recommended taxation, labeling e-cigarette devices with more detailed neurotoxic effects, and preventing their sale to pregnant women and adolescents.

## Figures and Tables

**Table 1:** Primer sequences chosen for quantitative real-time polymerase chain reaction analysis.

Gene	Name	NCBI ID#	Forward	Reverse
*Slc1a2 (GLT1)*	Glial high affinity glutamate transporter)	NM_001077514.4	5′GCA CGA GAG CTA TGG TGT ATT3 A3′	5′CTG CTT GAG TTT GGG ATT G3′
*mGLU2*	Metabotropic glutamate receptor 2	NM_001160353.1	5′CGC TAC AAC ATC TTC ACC TAT CT3′	5′AGT ATC CAG AGT CAG ACC TTC T 3′
*mGLU3*	Metabotropic glutamate receptor 3	NM_181850.2	5′CCA TGT GAG CCC TAT GAA TAC C3′	5′GGA AGG TTG TAG CAT CCA GAT AG3′
*Gapdh*	Glyceraldehyde-3-phosphate dehydrogenase isoform 1	NM_001289726.1	5′GTG GCA AAG TGG AGA TTG TTG3′	5′CGT TGA ATT TGC CGT GAG TG3′

## Data Availability

Data are available upon reasonable request.
